# A Novel Sparse Compositional Technique Reveals Microbial Perturbations

**DOI:** 10.1128/mSystems.00016-19

**Published:** 2019-02-12

**Authors:** Cameron Martino, James T. Morton, Clarisse A. Marotz, Luke R. Thompson, Anupriya Tripathi, Rob Knight, Karsten Zengler

**Affiliations:** a Department of Pediatrics, University of California San Diego, La Jolla, California, USA; b Bioinformatics and Systems Biology Program, University of California San Diego, La Jolla, California, USA; c Department of Computer Science and Engineering, University of California San Diego, La Jolla, California, USA; d Department of Biological Sciences and Northern Gulf Institute, University of Southern Mississippi, Hattiesburg, Mississippi, USA; e Ocean Chemistry and Ecosystems Division, Atlantic Oceanographic and Meteorological Laboratory, National Oceanic and Atmospheric Administration, stationed at Southwest Fisheries Science Center, La Jolla, California, USA; f Center for Microbiome Innovation, University of California San Diego, La Jolla, California, USA; g Department of Bioengineering, University of California San Diego, La Jolla, California, USA; University of Waterloo

**Keywords:** compositional, computational biology, matrix completion, microbiome, metagenomics

## Abstract

The central aims of many host or environmental microbiome studies are to elucidate factors associated with microbial community compositions and to relate microbial features to outcomes. However, these aims are often complicated by difficulties stemming from high-dimensionality, non-normality, sparsity, and the compositional nature of microbiome data sets. A key tool in microbiome analysis is beta diversity, defined by the distances between microbial samples. Many different distance metrics have been proposed, all with varying discriminatory power on data with differing characteristics. Here, we propose a compositional beta diversity metric rooted in a centered log-ratio transformation and matrix completion called robust Aitchison PCA. We demonstrate the benefits of compositional transformations upstream of beta diversity calculations through simulations. Additionally, we demonstrate improved effect size, classification accuracy, and robustness to sequencing depth over the current methods on several decreased sample subsets of real microbiome data sets. Finally, we highlight the ability of this new beta diversity metric to retain the feature loadings linked to sample ordinations revealing salient intercommunity niche feature importance.

**IMPORTANCE** By accounting for the sparse compositional nature of microbiome data sets, robust Aitchison PCA can yield high discriminatory power and salient feature ranking between microbial niches. The software to perform this analysis is available under an open-source license and can be obtained at https://github.com/biocore/DEICODE; additionally, a QIIME 2 plugin is provided to perform this analysis at https://library.qiime2.org/plugins/deicode/.

## Abstract (precis)

By accounting for the sparse compositional nature of microbiome data sets, robust Aitchison PCA can yield high discriminatory power and salient feature ranking between microbial niches. The software to perform this analysis is available under an open-source license and can be obtained at https://github.com/biocore/DEICODE; additionally, a QIIME 2 plugin is provided to perform this analysis at https://library.qiime2.org/plugins/q2-deicode.

## INTRODUCTION

Beta diversity is an ecological concept that describes differentiation in taxonomic or phylogenetic composition between communities. Beta diversity methods are a major component of many microbiome statistical analysis pipelines. These analyses enable an overview of complex microbial communities, identifying environmental factors differentiating microbial communities. However, there are dozens of distance metrics available to microbial ecologists to analyze their data, with each distance metric tailored to capture specific data characteristics. Beta diversity plots can therefore look dramatically different depending on the distance metric chosen, contributing to differences in interpretation of raw data (1).

One major confounding factor in beta diversity analysis is that microbiome data sets are sparse (i.e., most microorganisms are not found in most data sets), which has been shown to give rise to spike and horseshoe patterns in ordination plots (2, 3), complicating analysis. Furthermore, principal-component analysis (PCA) has common assumptions of normally distributed and linearly related variables, often violated by biological data (4–7). As a result, classical distance metrics that take into account only the presence/absence of taxa, such as the Jaccard index, or metrics that explicitly account for relative abundances, such as Bray-Curtis symmetrized distance, are commonly used. Microbial beta diversity estimation was greatly improved with the incorporation of phylogenetic information, as was shown with UniFrac (8), which can be used as either a presence/absence (unweighted) or relative abundance (weighted) metric. However, presence/absence methods often yield substantial differences between communities that are obscured by abundance-based methods. This might seem paradoxical, because abundance-based methods are integrating more information about the community. However, if the key players are rare rather than abundant species, or if abundant species display large random fluctuations, abundance information may obscure rather than clarify the result, even with phylogenetic metrics (9).

**FIG 1:**
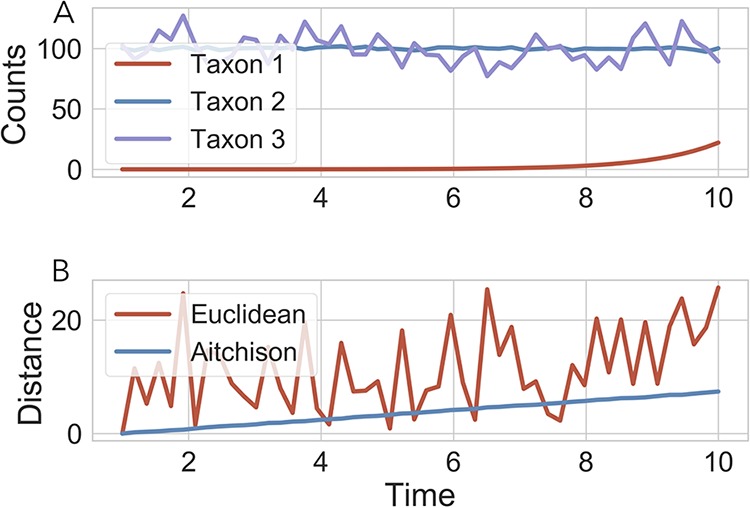
Benchmarking the rclr preprocessing step. Toy example with simple 3-taxon community sampled over time (A). Distance calculated between the *t* = 1 community and subsequent communities demonstrates the robustness of Aitchison distance compared to Euclidean distance (B).

Failure to reveal associations between phenotypes and the microbiome overall may also be a symptom of methods that do not properly account for the relative changes of microbial taxon abundances. To demonstrate this principle, consider the scenario in Fig. 1A, where three taxa are simulated over time. In this scenario, taxon 1 has a much lower abundance than the other two taxa, but it is growing exponentially over time. Taxon 2 has a high abundance and is stable over time. Taxon 3 also has a high abundance but fluctuates randomly. The Euclidean distance between the first community and the other two time points is extremely variable and does not capture the change induced by the exponential growth of taxon 1. This variability in the Euclidean distance is largely driven by the random fluctuations in the high-abundance taxa.

In contrast to Euclidean distance, compositional distance metrics, such as the Aitchison distance (see equation 2), can properly account for such relative changes (10). Here, the Aitchison distance factors in only the log fold change, reflecting the fact that deviations in the high-abundance taxa are large on an absolute scale but small on a relative scale. The difference between 100 counts and 120 counts is 20 counts, which is large compared to the abundance of the first taxon but is only a 20% increase. In contrast, the first taxon increased around 2,000%, and as a result, the Aitchison distance is driven by the large relative changes, including changes in the low-abundance species.

Microbes that display large fold change across samples will be weighted more heavily in the calculation of the Aitchison distance. However, this distance metric cannot handle zeros and is thus challenging to apply to the sparse data sets that characterize microbiome studies. There are many potential processes that could give rise to zeros in microbiome data. It is possible that there was undersampling, where low-abundance microbes were not detected in the sequencing data. Another possibility is that due to the heterogeneity of the sample, the microbe was not detected, even though it is present in the environment. Furthermore, it could be possible that the microbe is not present at all in the environment. In light of all of these potential processes, it is not feasible to differentiate between these different processes from the sequencing data (11, 12). To circumvent this issue, we propose a novel, compositional distance metric that can also explicitly handle sparse data through the use of matrix completion. This is done by treating all zeros as missing values and building a model to handle this missing data using matrix completion.

Matrix completion was originally developed in the context of recommender systems to predict user-item ratings (13) as a natural solution for handling sparse data. For example, the Netflix database contains a matrix detailing all customers by all movies where the entries are the movie ratings. However, each user rates only a small portion of the possible movies available on Netflix, so that only about 1% of the database contains nonzero values. As a result, when trying to recommend specific movies to specific customers, models need to be trained on the available ratings that customers have provided. Matrix completion tasks have become one of the state-of-the-art methods for performing these sorts of tasks.

Here, using simulation benchmarks and two case studies, we demonstrate the utility of preprocessing sparse microbiome data sets with matrix completion to allow compositional ordination and to preserve information about the features driving differences among samples.

## RESULTS

### Description of robust Aitchison PCA.

**FIG 2:**
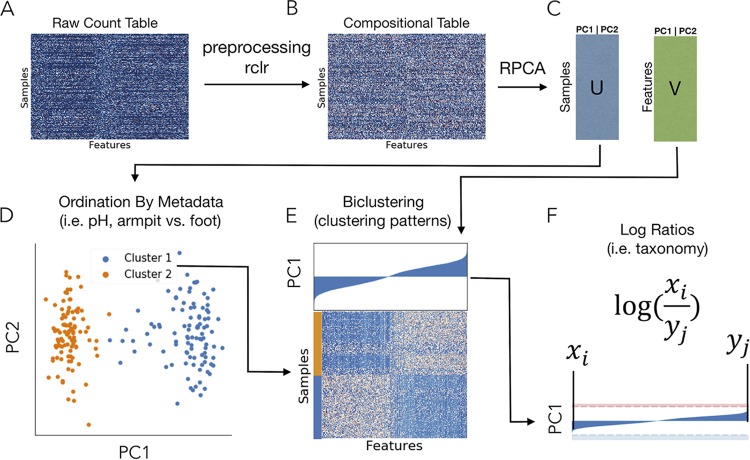
A general overview of the workflow. (A) A sparse, raw sequencing count table with samples on the *y* axis and features (i.e., OTUs, genes) on the *x* axis. (B) The data are preprocessed by a robust centered log ratio transform (rclr) on only the known (nonzero) values. (C) Matrix completion with a robust principal-component analysis (RPCA) that operates on only the observed values in the table resolves a loading by samples and by features. These loadings can be directly used for ordination (D), biclustering (E), and the identification of important taxa driving clustering in both the previous plots (F).

Matrix completion can be interpreted as a robust dimensionality reduction technique, where PCA is performed accounting only for the observed entries (i.e., ignoring the zeros). Matrix completion relies on two major assumptions. First, it assumes that data are missing at random, meaning that the missing entries in the matrix are uniformly distributed. Second, because matrix completion is a robust form of PCA, it assumes that the data are normally distributed and centered around zero (14). To meet this assumption, a commonly applied approach is to subtract the row and column means (15, 16). However, because microbiome sequencing data are represented as counts (17), the data are strictly positive and skewed toward zero, which confounds PCA. A workaround is to first log transform the nonzero values before centering the data—we will refer to this preprocessing procedure as the robust centered log ratio (rclr) due to its links to the centered log ratio (clr) transform commonly used in compositional data analysis (10) (Fig. 2A and B). A similar procedure using interquartiles was suggested previously (18).

This procedure produces a transformed table with missing values that can be used as input for matrix completion, or robust principal-component analysis (RPCA), which provides the sample and feature loadings. These sample and feature loadings contain the ordination information directly used in beta diversity plotting and feature biclustering (Fig. 2C to E). Because PCA preserves feature information, we can use the feature loadings to determine which taxa drive the differences among sample types (Fig. 2F).

### Simulations.

To benchmark the effectiveness of the rclr preprocessing step, we generated simulations from a study comparing microbial communities on keyboards and human fingertips (keyboard data set) (19) (see Materials and Methods for details). Simulated data were chosen as an initial proof-of-concept benchmark due to the ease of changing data set characteristics across which to interrogate; here the primary focus was on sequencing depth.

The simulated data were generated with two clusters over various sequencing depths from 1,000 to 10,000 reads per sample. At each sequencing depth, the output of the RPCA with and without the rclr transformation was compared by Kullback-Leibler divergence (KL) (20) to the simulation ground truth between rclr preprocessed and raw count data. Additionally, ordination output was compared by permutational multivariate analysis of variance (PERMANOVA) F-statistic and supervised k-nearest neighbor (KNN) classification cross-validation (40:60) split.

**FIG 3:**
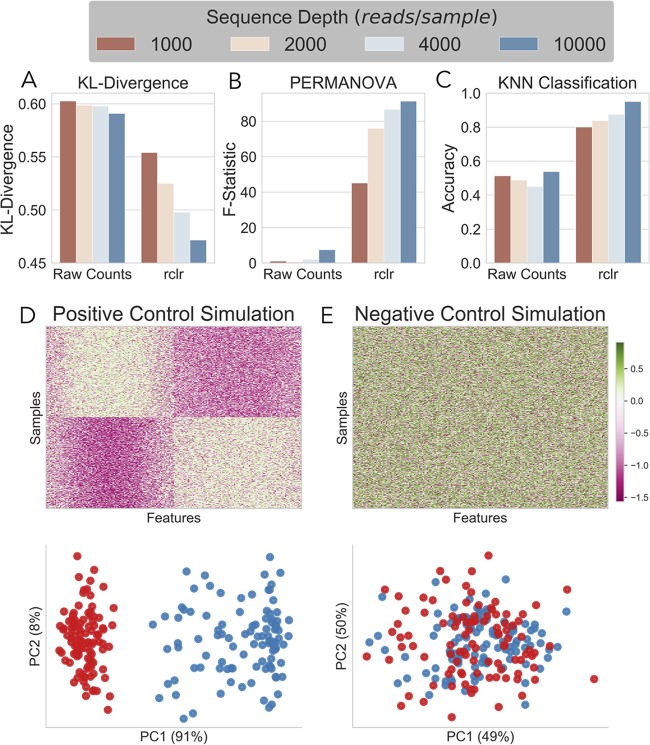
(A) Comparison of KL-divergence (*y* axis) between simulated base truth data between RPCA output from raw count data and rclr-preprocessed data. (B and C) Comparison between RPCA ordination by PERMANOVA F-statistic (B) and KNN classifier accuracy (C). All are at various sequencing depths from 1,000 to 10,000 reads per sample. (D and E) Comparison of positive- (D) and negative-control (E) simulation by biclustering (top) and RPCA ordination (bottom).

When rclr preprocessing was applied, we saw a decrease in mean KL, demonstrating a more closely matched probability distribution when using the rclr (Fig. 3A). Furthermore, when the rclr was applied, the F-statistic demonstrated a 4-fold increase (Fig. 3B) and KNN classification accuracy (Fig. 3C) increased by between 30 and 40%. All of the metrics, when applied to rclr RPCA, improved as the sequencing depth improved, following the logic that a good fit should increase performance as sequencing depth increases. A negative-control simulation with no group discrimination revealed no biclustering, RPCA clustering (Fig. 3E), low KNN classification accuracy, and PERMANOVA significance compared to a positive control (Fig. 3D) with two distinct groups (see Table S1 in the supplemental material). This demonstrates a proof of concept that rclr is less affected by outliers and is reliably reproducible at low and high sequencing depths.

10.1128/mSystems.00016-19.4 TABLE S1 Comparison of PERMANOVA and KNN classifier accuracy between positive- and negative-control simulations. Download Table S1, XLSX file, 0.01 MB. Copyright © 2019 Martino et al. 2019 Martino et al. This content is distributed under the terms of the Creative Commons Attribution 4.0 International license.

### Case studies.

Next, we demonstrated the utility of RPCA compared to the current state of the art. To do this we used two 16S rRNA gene amplicon sequencing data sets. The first data set is a subset of the Sponge Microbiome Project (sponges) (21), where we compared sponge microbial communities classified by health status (i.e., stressed or healthy). The second data set derives from a sleep apnea study; it consists of mouse fecal samples and focuses on comparing the gut microbiome of animals exposed to intermittent hypoxia and hypercapnia (IHH; as a model of obstructive sleep apnea) to controls exposed to room air (air) (22).

**FIG 4:**
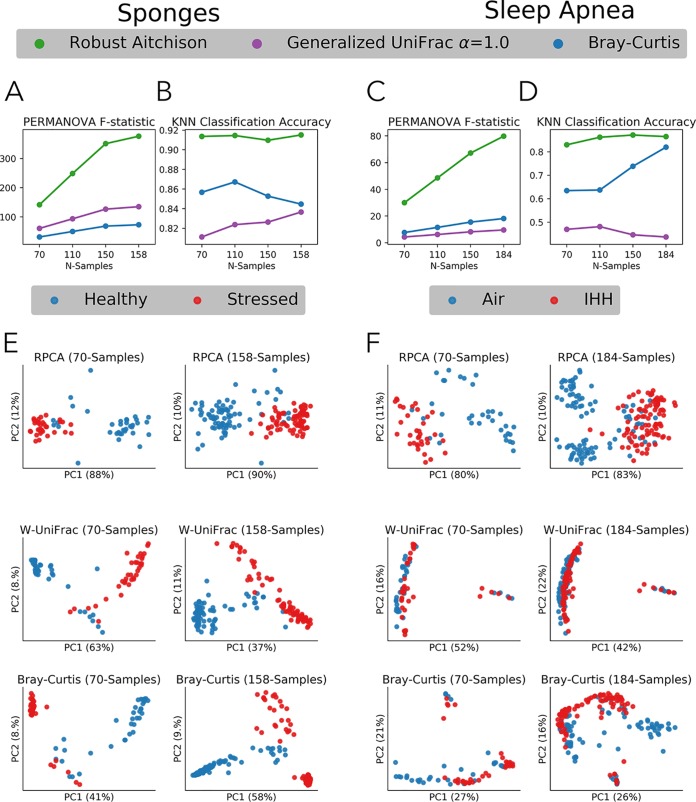
A case study of RPCA on real data sets; sponge (left; A, B, and E) and sleep apnea (right; C, D, and F). PERMANOVA F test statistic (*y* axis) (A and C) or KNN classifier accuracy (B and D) by subsamples of the data sets. Ordination plots between 70 samples total (left) and maximum number of samples (right) compared between RPCA (top), generalized weighted UniFrac (alpha = 1) (middle), and Bray-Curtis (bottom) (E and F). Sponge data set plotted between healthy (blue) and stressed (red) (E) along with sleep apnea data set plotted between air (blue) and IHH (red) (F).

Many different metrics exist for beta diversity distance comparison. We compared RPCA to two of the most commonly employed abundance-based methods, Bray-Curtis and weighted UniFrac, over 10-fold random subsamples of the data. The distances between the highlighted metadata categories for the two data sets were compared over subsamples with PERMANOVA (Fig. 4A and C). The principal coordinate analysis (PCoA) was compared by supervised KNN classification cross-validation (40:60 split) accuracy for both data sets over subsamples (Fig. 4B and D). In all subsample comparisons, the robust Aitchison (distance metric derived from RPCA) outperformed Bray-Curtis and weighted UniFrac. The results are qualitatively demonstrated in the PCoA clustering between metadata categories for low and high subsample depths (Fig. 4E and F).

A key benefit of RPCA over metrics, such as weighted UniFrac and Bray-Curtis, is direct access to the feature loadings. With Euclidean distance it is also possible to obtain feature loadings. However, Euclidean distance has multiple undesirable properties, such as artifacts in clustering patterns and weak discrimination in high-dimensional sparse data (2, 6, 7, 23, 24), making the values unreliable. Fortunately, feature loadings can be extracted from RPCA while utilizing the same interpretation as suggested in the work of Aitchison and Greenacre (25). These feature loadings can be largely explained by a few features (26). This ultimately allows us to rank the taxa in the data in relation to the samples and the metadata. When sorted, often referred to as biclustering, this method results in a table that reveals which taxa are driving the clustering seen in the ordinations.

**FIG 5:**
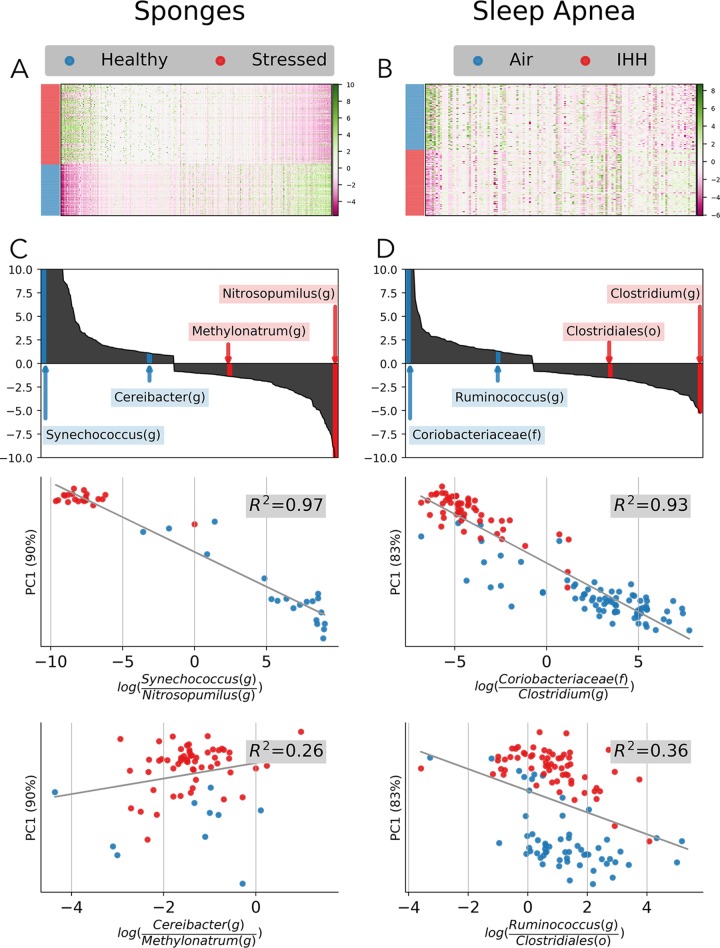
A case study of RPCA feature loadings on real data sets; sponge (left; A and C) and sleep apnea (right; B and D). Heat maps of clr-transformed sOTU tables with samples sorted by metadata and features sorted by RPCA feature loadings (A and B). Absolute highest (middle) and lowest (bottom) feature loading sOTUs (top) plotted as log ratios (*x* axis) by sample loading PC1 (*y* axis) (C and D).

In this case, we have a two-block table represented by clr-transformed heat maps for the sponges (Fig. 5A) and sleep apnea (Fig. 5B) data sets. It is evident from the heat map and ordination plots that there are some taxonomic abundance changes between the categories that are dividing the clusters. In order to compare two taxa directly, we applied log ratios on highly weighted features. The highest loaded features (most positively ranked and most negatively ranked) correspond to the most influential taxa driving the clustering. Interesting pairs of taxa were identified in the sponge data set (Fig. 5C) and the sleep apnea data set (Fig. 5D). These log ratios were shown to be correlated with the sample loadings in the PC1 axis (*R*^2^ = 0.97 and 0.93). To show that not all of the taxa were significantly contributing to the variation in PC1, two pairs of insignificantly ranked log ratios were also identified (*R*^2^ = 0.26 and 0.36).

The highly weighted log ratios in the sponge case study indicate that two sub-operational taxonomic units (sOTU) can explain a great deal of variation between healthy and thermally stressed sponges. The sOTUs most strongly associated with healthy and stressed sponges, respectively, were classified at the lowest assignment level to “*Candidatus* Synechococcus spongiarum” (species, numerator) and *Nitrosopumilus* (genus, denominator). Both of these groups are known sponge symbionts (27, 28). *Nitrosopumilus* is an ammonia-oxidizing archaeon, which nitrifies ammonia to nitrate. Nitrification by sponge-associated microbiota is thought to remove ammonia waste produced by the host sponge (27, 29). It has been proposed that ammonium, urea, and creatine leaking from host sponge tissue could promote growth of *Nitrosopumilus* (30), and this leakage may be more active in stressed hosts. “*Candidatus* Synechococcus spongiarum” has been found in numerous sponge species around the globe (28), and its photosynthetic products may contribute to host nutrition (31). From this analysis, this sOTU and several other sOTUs of “*Candidatus* Synechococcus spongiarum” (32) appear to be strongly associated with healthy sponges relative to stressed sponges.

In the sleep apnea data set, the highly weighted log ratios revealed a strong clustering of air versus IHH. These sOTUs were classified as *Coriobacteriaceae* (family) and *Clostridium* (genus). This trend was also observed by Tripathi et al. (22), where it was corroborated by the perturbations in the small molecular products attributed to members of these taxonomic classes. For example, changes in *Clostridium* were reflected in downstream changes in intestinal bile acids, as members of this genera are known to transform bile acids (33). Previous studies (34, 35) have also reported changes in these taxonomic classes in cardiometabolic comorbidities of sleep apnea, which suggests that our method potentially guides biologically relevant observations.

## DISCUSSION

Here we demonstrated the ability of rclr preprocessing and RPCA to reveal salient, beta diversity ordination and factor loading. We demonstrated through simulations that rclr preprocessing dramatically improved RPCA. In two case studies (sponge and sleep apnea), RPCA presented higher PERMANOVA F-statistics and KNN classifier accuracy in small subsamples of the data. In addition, RPCA qualitatively increased the discriminative ability of clusters obtained from the ordination over beta diversity techniques widely used in the field, at both low and high levels of subsampling.

We have shown that Aitchison distance has numerous other desirable properties, such as scale invariance, negating the need to perform rarefaction. This feature is critical when one lacks access to absolute microbial abundance, because scale invariant distances ensure equivalence between distances computed from absolute and relative abundance measurements (see Materials and Methods for equation). Aitchison distance is also known to be subcompositionally coherent (36). This guarantees that distances will never decrease if additional taxa are observed (e.g., by using PCR primers with broader specificity), which has important implications for reproducibility across distance-based analyses, especially across studies that use different molecular methods.

The increased cluster separation at smaller subsamples of the data set highlights the robustness of the method. Significant partitioning of sample categories on smaller sample cohorts is particularly important in a clinical setting, due to the difficulty of large-volume sample collection. In addition, rapid resolutions of taxa driving ordination are of principal importance in translational results.

Importantly, because RPCA provides linked sample and feature information, one can directly identify which taxa are likely driving sample clustering (which are typically separate workflows in canonical amplicon analysis). However, RPCA does not currently take into account phylogenetic relationships among features. Adapting phylogenetic ilr transforms (37) could potentially improve the resulting ordinations.

It is important to note that while there have been previous methods that handle zeros in compositional data sets, such as methods available in zCompositions (38), the methodology here is fundamentally different. First, the zeros in microbiome data sets are never factored into the computation. In addition, the matrix completion approach presented here is a natural solution to high-dimensional data sets, which are not currently addressed in methods available in zCompositions.

In light of these benefits, there are still challenges that need to be considered, namely, overfitting and the low-rank approximation. Given the high-dimensional nature of microbial data sets, the number of parameters required to fit robust principal components can grow very quickly. As a result, it is still possible to overfit these methods, making them potentially sensitive to outliers and reducing their predictive power (39), although we did not notice these effects in our simulations. We therefore recommend starting fitting RPCA models with a low rank of either two or three for microbiome studies containing approximately 100 samples. The rank can be increased if there are appropriately many samples.

A low-rank constraint can possibly cause misleading results in the case of high-rank data sets. High-rank data sets may occur in microbiome data sets as a gradient between samples and features. To give intuition of what types of data may contain high-rank structure, we provide two published examples. The first example is a study of soil microbiomes representing different pH environments (40) (see Materials and Methods for detail). The second example is a case study of the gut colonization of an infant over time (41) (see Materials and Methods for detail). In both cases, a gradient forms because very few samples contain similar microbes (see Fig. S1 in the supplemental material). For example, in the infant development study very few microbes are shared between subsequent samples over time. Although the rclr transform eases the problem, it can still lead to misinterpretation in ordination (see Fig. S2 in the supplemental material). There are many possible future directions for incorporating regularization or Bayesian priors to better fit these models.

10.1128/mSystems.00016-19.1 FIG S1 Sorted heat map plots of example gradient structured high-rank data sets for 88 soils (A) and infant development data set (B). Download FIG S1, TIF file, 1.7 MB. Copyright © 2019 Martino et al. 2019 Martino et al. This content is distributed under the terms of the Creative Commons Attribution 4.0 International license.

10.1128/mSystems.00016-19.2 FIG S2 Comparison of methods RPCA without rclr (A), RPCA with rclr (B), and Bray-Curtis (C) in high-rank infant development data set at various numbers of samples of 30, 40, and 50 from left to right. Download FIG S2, TIF file, 1.4 MB. Copyright © 2019 Martino et al. 2019 Martino et al. This content is distributed under the terms of the Creative Commons Attribution 4.0 International license.

In light of the current limitations, we have shown that matrix completion resolves numerous outstanding problems in beta diversity analysis, including sparsity, compositional effects, and uneven sequencing depths, all while giving information about the taxa driving microbial perturbations. This method could possibly be adapted to or combined with other omics paradigms (e.g., metabolomics, metatranscriptomics, and metagenomics) and provides the opportunity to initiate standardization of beta diversity analyses in the microbiome field.

## MATERIALS AND METHODS

### Preprocessing with rclr.

Prior to running matrix completion, the data need to be centered around zero and approximately normally distributed. The centered log ratio (clr) transformation is commonly applied in compositional data analysis before applying PCA. This log transforms each value and then centers them around zero. This is particularly useful when one assumes that the data are lognormally distributed as proposed in reference 42, since log-transformed lognormally distributed data are normally distributed. The clr transform is given below:

(1) $$\text{clr}\left(x\right)=\left[\log\frac{x_{1}}{g\left(x\right)}\text{,}\ldots \text{, }\log\frac{x_{D}}{g\left(x\right)}\right]=\log x-\overset{¯}{\log x}$$

where *g*(*x*) is the geometric mean of all of the taxa. The Aitchison distance can be directly calculated from the Euclidean distance of the clr-transformed data. This is given as follows:

(2) $$d_{A}\left(x,y\right)=\sqrt{\sum_{i=1}^{D}{\left(\text{clr}{\left[x\right]}_{i}-\text{clr}{\left[y\right]}_{i}\right)}^{2}}=\sqrt{\sum_{i=1}^{D}{\left(\log\frac{x_{i}}{x_{j}}-\log\frac{y_{i}}{y_{j}}\right)}^{2}}$$

The Aitchison distance between the absolute abundances is equivalent to the Aitchison distance on the proportions. In order to center the samples around zero, the average clr-transformed sample needs to be calculated and then subtracted from the remaining samples. Thus, the clr-transformed results will be as follows:

(3) $$y_{ij}=\log x_{ij}-\bar{\text{log}x_{i}}-\bar{\log x_{j}}$$

This centering procedure is commonly used prior to performing PCA and eliminates the need to explicitly compute bias constants (43).

The issue with applying the clr transform directly to sparse count data is that the log of zero is undefined. This motivated the construction of an approximate clr transform defined only on nonzero counts. The robust clr (rclr) transform is given as follows:

(4) $$\text{rclr}\left(x\right)=\left[\log\frac{x_{1}}{g_{r}\left[x\right]}\text{,}\ldots \text{,}\log\frac{x_{D}}{g_{r}\left[x\right]}\right]$$

(5) $$g_{r}\left(x\right)={\left(\prod_{i\varepsilon \Omega_{x}}^{}x_{i}\right)}^{1/|\Omega_{x}|}$$

where *x_i_* is the abundance of taxa *i*, Ω*_x_* is the set of observed taxa in sample *x*, and *g_r_*(*x*) is the geometric mean defined only on observed taxa. The rationale behind this procedure is that due to the high dimensionality of these data sets, the robust geometric mean (the geometric mean of the log-transformed nonzero data) can serve as an approximation to the true geometric mean. We know from the Central Limit Theorem that as we collect more independent measurements, we approach the true geometric mean:

(6) $$\frac{1}{\left|\Omega_{x}\right|}\underset{i\varepsilon \Omega_{x}}{\sum }x_{i}\to E\left[\log\overset{\to }{x}\right]\text{ as }|\Omega_{x}|\to |\overset{\to }{x}|$$

From this we can redefine the transformed result as follows:

(7) $$y_{ij}=\;\log x_{ij}-\frac{1}{\left|\Omega_{x_{i\text{.}}}\right|}\overset{}{\underset{k\varepsilon \Omega_{x_{i.}}}{\sum }}x_{k}-\frac{1}{\left|\Omega_{x_{.j}}\right|}\overset{}{\underset{i\varepsilon \Omega_{x_{.j}}}{\sum }}x_{k}$$

where *y_ij_* is only defined when *x_ij_* > 0. The matrix completion methods can then be directly applied to this transformed result.

### Matrix completion.

OptSpace is a matrix completion algorithm based on a singular value decomposition (SVD) optimized on a local manifold. It has been shown to be quite robust to noise in low-rank data sets (44). The objective function that it optimizes over is given by

(8) $$\underset{{}_{U,V}}{\min}{\left|\Lambda [Y-\mathrm{US}V^{T}]\right|}_{2}^{2}$$

where *U* and *V* are the matrices that are trying to be estimated and *S* is analogous to a matrix of eigenvalues. *Y* is the observed values, and Λ is a function such that the errors between *Y* and *USV^T^* are computed only on the nonzero entries.

### Simulations.

Simulations were designed to replicate real data sets with low-rank clusters as a proof-of-concept test of OptSpace with and without the rclr preprocessing step. The keyboard data set was chosen as a representative data set to fit the simulation parameters due to the three distinct microbial community clusters observed in the study (M2, M3, and M9). Simulations were built by drawing blocks of *n* sequences with the microbial proportions given as follows (45):

(9) $$x_{ij}=\frac{1}{\sqrt{2{\text{πσ}}^{2}}}\text{exp}\left(\frac{{\left[{\text{μ}}_{i}-g_{j}\right]}^{2}}{2{\text{σ}}^{2}}\right)$$

(10) $$p_{ij}=\frac{x_{ij}}{\Sigma_{k}x_{kj}}$$

The resulting simulation was induced by multiple noise sources. There was normally distributed error that was applied to the entire matrix. There were also normally distributed errors that were randomly applied to a subset of the entries in the matrix. In addition, there were subsampling errors that were simulated from the Poisson-lognormal (PLN) distribution with an overdispersion parameter ϕ (46) where the final subsampled simulation *y_ij_* is represented by:

(11) $${\text{λ}}_{ij}=np_{ij}$$

(12) $$y_{ij}=\text{PLN}\left({\text{λ}}_{ij},\phi \right)$$

The resulting optimized parameters are optimized rank (number of clusters), the intensity of noise, sequencing depth, the distribution parameters μ and σ, and overlap of features between clusters (i.e., effect size). To resolve the most realistic simulation possible, these parameters were optimized to minimize the KL-divergence between the real data and the simulation with a Broyden-Fletcher-Goldfarb-Shanno (BFGS) optimization. The resolved parameters were used to run the simulation at a rank of 2 over sequencing depths ranging from 100 to 10,000 reads/sample. At each depth, before the introduction of noise and subsampling, the sampled data were stored as a base truth to be compared to the reconstruction. Furthermore, the same noisy and subsampled simulation was run with OptSpace with or without rclr preprocessing. The resulting matrix *USV^T^* was compared by KL-divergence to the base truth. The rclr-preprocessed data were inverse transformed by taking the exponential of *USV^T^* before comparison to the base truth. In addition, the simulation, base truth, sample orientation *U*, and feature loadings *V^T^* were saved at each iteration and compared visually.

The simulation results of improved clustering at uneven sequencing depths were also compared in the real keyboard data set (see case studies for data processing). The data were compared between two subjects at 500 and 100 reads/sample. Ordination and PERMANOVA results were compared for Jaccard, Bray-Curtis, and RPCA with rclr preprocessing. RPCA with rclr preprocessing alleviated the clustering by sequencing depth in the real data set. This was seen both qualitatively (see Fig. S3 in the supplemental material) and through the PERMANOVA F-statistic by subject ID (see Table S2 in the supplemental material).

10.1128/mSystems.00016-19.3 FIG S3 Comparison between two subjects (green and blue) from the keyboard data set by sequencing depth 500 (circles) and 100 (squares) over different beta diversity methods, Jaccard (A), Bray-Curtis (B), and RPCA (C). Download FIG S3, TIF file, 1.2 MB. Copyright © 2019 Martino et al. 2019 Martino et al. This content is distributed under the terms of the Creative Commons Attribution 4.0 International license.

10.1128/mSystems.00016-19.5 TABLE S2 Comparison of PERMANOVA F-statistic and *P* value between subject ID clusters in the keyboard data set with uneven sequencing of 500 and 100 reads/sample. Download Table S2, XLSX file, 0.01 MB. Copyright © 2019 Martino et al. 2019 Martino et al. This content is distributed under the terms of the Creative Commons Attribution 4.0 International license.

### Case studies.

Case studies on real-world data sets were used to compare robust Aitchison PCA to the current state of the art in beta diversity comparison. The sponge, sleep apnea, infant, keyboard, and 88-soil data sets were acquired on 20 September 2018 from Qiita (47) with IDs of 10793, 10422, 101, 232, and 103, respectively. Each data set was run through Qiita with default trimming and Deblur (v. 1.1.0) sOTU (48) picking approach, using QIIME 2 (v. 2018.6.0) (49). The resulting BIOM (50) tables were then filtered for samples greater than 1,000 reads per sample. Phylogeny was built using the most up-to-date GreenGenes using SEPP (51), and taxonomy was assigned through scikit-learn with default QIIME 2 parameters.

The sponge data set was filtered using the metadata so that it contained only samples with either the label healthy or the label stressed. This resulted in a comparison with 218 remaining samples. Similarly, the sleep apnea study was filtered for IHH and air control samples, with a treatment duration of 6 weeks resulting in 189 remaining samples. The infant gut colonization case study was filtered for samples over 500 reads/sample and for a single sample from the mother with the title 101.Mother. The 88-soil data set was filtered for samples over 500 reads/samples. The keyboard data set was filtered for samples over 500 reads/sample and 15 reads/sOTU. Additionally, only subject IDs corresponding to M3, M2, and M9 were retained, giving 67 samples. For comparing ordinations at different numbers of samples, the data sets were filtered for having 1,000 sequences/sample and balanced to have equal numbers of each subgroup (i.e., equal Air and IHH samples). Then samples were removed randomly but equally from each subgroup; this was repeated 10 times. The first iteration was used to plot the ordinations, and the mean score of the iterations was used to plot KNN classification accuracy and PERMANOVA F-statistic.

Both data sets were then preprocessed with the robust centered log ratio (rclr) transform, and RPCA was run with a rank of 2 because there were two metadata categories of interest in each comparison. Weighted UniFrac distances were calculated using generalized UniFrac with an alpha of one (52). Bray-Curtis distances were calculated through QIIME 2 (49). Both weighted UniFrac and Bray-Curtis distances were calculated on tables rarefied to 1,000 reads per sample. PCoA and PERMANOVA analyses for the Bray-Curtis, RPCA distance matrix, and weighted UniFrac were calculated through scikit-bio. The resulting PCoA and PCA axes were plotted through matplotlib (53) with PC1 and PC2 in the *x* and *y* axes, respectively.

The original unprocessed (raw count) tables were sorted by feature loadings from RPCA. Features with a count sum of less than 10 across all samples were filtered out. The resulting table was then clr transformed with a pseudocount of one and plotted as a heat map. Each sOTU was given the lowest classification for the sleep apnea and sponge data sets, respectively.

The features in the PC1 axis of the feature loadings from RPCA were selected to represent a manageable number of taxa to compare between subgroups. Those selected features (sOTUs) from the feature loadings were used for log ratios. Log ratios were calculated from the table used to calculate them. The samples that contained zeros in either the numerator or denominator were removed before calculating the ratios. The correlations between the log ratio and PC1 axis were performed by Pearson correlation via SciPy (54).
